# Does Internet use connect smallholder farmers to a healthy diet? Evidence from rural China

**DOI:** 10.3389/fnut.2023.1122677

**Published:** 2023-04-20

**Authors:** Mingwei Yang, Zhiyong Zhang, Zheng Wang

**Affiliations:** ^1^Research Center for Economy of Upper Reaches of the Yangtse River, Chongqing Technology and Business University, Chongqing, China; ^2^Qinghai University Library, Qinghai University, Xining, Qinghai, China; ^3^School of Economics and Management, Taiyuan Normal University, Taiyuan, China

**Keywords:** dietary diversity, dietary rationality, rural residents, Internet use, smallholder farmers

## Abstract

**Introduction:**

Undernutrition and micronutrient malnutrition remain problems of significant magnitude among small-scale subsistence farmers, posing a serious threat to their health and well-being. Developing a healthy diet can effectively reduce this threat. Fortunately, the Internet can speed up the process.

**Methods:**

Based on survey data from 5,114 farm households in nine provinces in China, this study quantitatively assesses the impact of Internet use on the dietary quality of smallholder farmers using OLS regression models and PSM models.

**Results/Discussion:**

(1) Internet use can significantly contribute to dietary diversity and dietary rationality among smallholder farmers, thus optimizing their dietary structure. (2) Internet use significantly increased the average consumption amounts of milk and its products (2.9 g), fruits (21.5 g), eggs (7.5 g), and vegetables (27.1 g), while also decreasing the intake of salts (1.5 g) and oil (3.8 g). (3) The pull of internet use to improve diet quality is more significant for smallholder households with lower levels of education, older heads of households, and higher household incomes. (4) A possible mechanism is that Internet use increases household income and information access skills of rural residents, thus improving their dietary quality. In summary, governments should further promote Internet penetration in rural areas for health purposes.

## 1. Introduction

In the digital economy, people’s diets and nutritional profiles have changed dramatically. People have begun to pay more attention to their health and seek healthy and nutritious food (1). However, there are obvious contradictions between people’s dietary aims and dietary behaviors, especially in rural areas. For example, in rural areas of China, small-scale subsistence farmers have continued traditional dietary habits (e.g., high-carb, high-salt, high-sugar diets and long-term consumption of food at night). Such a diet may greatly impact the welfare of rural residents, leading to, for example, increased disease, increased medical burden, etc., and thus undermining rural human resource development, and further threatening the sustainable development and revitalization of rural areas (2).

Many of the undernourished people in China are small-scale subsistence farmers. In 2021 China’s arable land *per capita* is 0.097 hectares, far below the world average for arable land *per capita* (3). Smallholder farmers in China are considered to be disadvantaged in terms of economic, human and information capital due to the shortage of arable land resources *per capita* (4, 5). For a long time, the lack of *per capita* arable land resources in rural China has resulted in a relatively homogeneous diet for Chinese farmers, which is often limited to food harvested from their own farms. At the same time, smallholder families are more isolated from information, have poor access to food, and are economically disadvantaged, all of which are important reasons for the backward structure of diets and dietary attitudes in rural China. Specifically in terms of nutritional status, a basic manifestation of smallholder households is poor dietary patterns. Smallholder farmers ‘diets are mostly home-harvested or wild-harvested. The primary requirements for food are good taste, easy to cook, and cheap ingredients, but little consideration is given to whether the food contains nutrition and how much nutrition it contains. For a long time, many rural areas lacked adequate meat, many farmers believe that a good meal means eating enough meat, and eating vegetables is to treat oneself poorly, and they do not know what a healthy diet is. The shortage of meat in the past has led to a preference for carnivorous foods among Chinese farmers. In recent years, as the economy and income levels in rural areas have risen, the living conditions of farmers have improved considerably. However, the preference for meat-based foods still persists. At the same time, there is still a large number of poor people in rural China and malnutrition is widespread. The skyrocketing incidence of chronic diseases among Chinese rural residents also indicates that the dietary structure of farmers is highly problematic.

According to the Report on Nutrition and Chronic Diseases in China (2020), the fat to energy ratio of Chinese farmers continues to increase, with the rate of over-weight and obesity among adults exceeding 30% and the incidence of food-borne chronic diseases such as diabetes increasing year on year (6). Dietary risks closely related to overweight and obesity have become the number one mortality factor for Chinese farmers (7–9). However, due to economic constraints and lack of dietary health awareness, a large number of farmers have continued traditional dietary habits (e.g., high-carb, high-salt, high-sugar diets, and long-term consumption of food at night) (10, 11). Such a diet may greatly impact the welfare of rural residents, leading to, for example, increased disease, increased medical burden, decreased human capital, etc., and thus threatening the sustainable development and revitalization of rural areas (12–14).

The data from the current research encourage facilitating a relationship between the existing Internet technology and dietary health. First, farmers can access information on food and healthy diets through the Internet, thus changing their dietary choice preferences and promoting the upgrading of dietary structure (15–17). Second, the digital economy has greatly facilitated the consumer experience by providing services to farmers in areas, such as finance, online shopping, and social networking, enabling farmers to access quality food and services without discrimination (18, 19).

Researches on the impact of the Internet and the digital economy on food consumption provides an important reference for this study, but some issues remain unclear. For instance, some scholars believe that increased income does not mean improved dietary structure (20, 21). Traditionally, many farmers believe that a good diet means eating enough meat, and eating vegetables is to treat oneself poorly. Their basic requirements for food are good taste, easy to cook, and cheap ingredients, and little consideration is given to whether the food contains nutrition and how much nutrition it contains. China’s rural residents face a variety of nutritional health problems caused by an unreasonable dietary structure, which not only affects the overall quality of the population, but also brings a heavy medical burden and high economic costs (22–24). Therefore, better meeting the growing needs of the people for a better life objectively requires intensified research on the upgrading of the dietary structure of farm households.

In addition, the development of digital technology has also created new technical barriers and does not provide equal opportunities for farmers to share in the digital dividend (25–28). The relatively low level of human capital in rural areas and the barriers to technology use in the Internet process faced by farming households, especially the large number of middle-aged and elderly people in rural areas who generally face barriers to digital technology use (29, 30). Therefore, it makes sense to analyze the heterogeneity of Internet use.

With economic development and income growth, Chinese residents’ consumption of starchy staple foods such as rice and flour will gradually decrease, but consumption of nutrient-rich meat, vegetables and fruit will increase significantly (31). China is about to cross the threshold of a high-income country and the upgrading of its consumption structure will accelerate in the future (32–34). Food ration consumption is already around the peak and food consumption of meat, eggs and milk, fruits, and vegetables are still rising, and the dietary structure of Chinese residents is undergoing great changes (35). In this context, this paper mainly answers the following questions based on the survey data from 5,414 farm households: (1) Does Internet use promotes smallholder farmers’ dietary quality? (2) What is the underlying mechanism involved? (3) Are the improving effects on dietary structure heterogeneous?

There are two contributions of this study. First, in terms of the research topics, this paper quantitatively assesses the impact of Internet use on the dietary quality of smallholder farmers. Many of the undernourished people in rural areas of China are small-scale subsistence farmers. And smallholder farmers are disadvantaged in terms of land capital, economy capital and information capital, exploring the impact of Internet use on the dietary quality of smallholder farmers helps to extend research on rural lifestyle changes in the digital age. Second, the possible mechanism—that Internet use enhances smallholder farmers’ household incomes and information access skills—was empirically tested, which could reveal more implications.

The remainder of the paper is organized as follows: Section 2 proposes a theoretical analysis and three research hypotheses. Section 3 introduces the identification strategy, variables, and data for this study. Section 4 tests two hypotheses and presents the regression results and covers the heterogeneity analysis and robustness testing. Section 5 provides the discussions, conclusions and related policy implications.

## 2. Theoretical analysis and research hypothesis

Along with the rapid development of the digital economy and the extensive embedding of digital technologies, China’s rural socioeconomic sectors are undergoing profound digital transformation (36–38). Internet is not only a key element in enhancing production efficiency, but has also become an important driving force in upgrading the structure of farmers’ diets (39, 40). In the context of the increasing capacity of rural dwellers to use digital technology, digital skills have an impact on the structure of farm households’ diets mainly through the following pathways.

Internet use can raise the income levels of farming households and relax economic constraints, thus improving the structure of diets. The income-generating effect of digital skills is mainly reflected in three aspects. Firstly, Internet use can enhance the marketing ability of farmers’ products and increase their business income (41–43). The use of information and communication technology has promoted the specialization of crop cultivation, significantly improving the efficiency of the choice of marketing channels for farmers, helping to expand the market reach and selling price of agricultural products, and greatly enhancing the profitability of agricultural operations and non-farm operations (44–46). Secondly, Internet use can increase non-farm employment opportunities for farmers and improve their wage income (47, 48). The Internet has the advantage of low learning and training costs, and the use of digital technology and the acquisition of digital skills helps to broaden the non-agricultural employment options of the rural workforce and enhances job matching, increasing their employment and formal employment rates and strengthening employment stability (49–51). Thirdly, Internet use can facilitate asset factor allocation and increase property income (52, 53). Internet use has increased the scale of financial assets of farm households, helping to improve the asset allocation structure and promoting higher property income for farm households (53–55). In short, Internet use can increase the level of operational income, wage income and property income of farming households, thus increasing total household income. At the same time, the increase in income level has an important role in promoting the upgrading of dietary structure. Some studies have pointed out that income growth has a significant positive impact on energy, fat, and protein consumption of farm households, which is conducive to residents reducing the consumption of low-quality food and increasing the consumption of high-quality food, thus promoting the upgrading of dietary structure (56–58). Therefore, farmers can improve their dietary structure in terms of food quality and quantity by increasing their income level, and promote the growth of nutrient intake, especially by increasing the consumption of food with higher nutritional value such as dairy products to ensure the transformation and upgrading of food consumption structure and balanced nutritional intake, thus achieving the upgrading of dietary structure (59).

Internet use can ease farmers’ information constraints and thus improve dietary structure (60–62). First, Internet use can expand farmers’ access to information on healthy diets and reduce information asymmetry, as well as strengthen farmers’ awareness of investing in health, helping them to develop a healthy diet, increase their preference for foods with high nutritional value and improve their dietary structure (63, 64). Secondly, Internet use can provide convenient food consumption information services and increase the availability of high-quality food to farmers (65–67). E-commerce, cold chain, and courier services meet farmers’ food consumption needs for safety, freshness, and timeliness and facilitate the availability of high nutritional value foods such as meat, eggs and milk to farmers (68–70). In addition, the development of the Internet has weakened consumer budget constraints by reducing commodity prices and transaction costs, among other paths, in favor of higher spending on quality food by farming households (71, 72).

Rural e-commerce refers to the process of using the Internet, computers, and other modern information technology to provide production and business entities engaged in agriculture-related fields to complete business transactions such as the sale, purchase and electronic payment of products or services online. In this paper, rural e-commerce refers to the sale of farmers’ own agricultural products to increase their income on the one hand, and also denotes farmers’ enhanced access to food through online shopping on the other. Both of these aspects have important implications for the meal patterns of smallholder farmers. In short, Internet use can provide rural residents with more convenient and diversified services and products, making it possible and convenient for them to upgrade their dietary structure. Based on the above analysis, we propose Hypotheses 1, 2.

It is worth noting that, limited by the availability of data, there is a correlation between Internet use—enhanced household income/information access—improved dietary quality (dietary diversity and dietary rationality) among smallholder farmers, which is the focus of this paper. The heterogeneity and mechanism analyses in this paper confirm the existence of this correlation. However, there may be a causal relationship between household income/information access and Internet use, which needs to be confirmed by more empirical evidence.

*H1*: There is a significant positive relationship between Internet use and dietary diversity and dietary rationality in smallholder households.

*H2*: The positive association between Internet use and meal quality among smallholder households stems mainly from increased access to information and relaxation of economic constraints.

**Figure 1:**
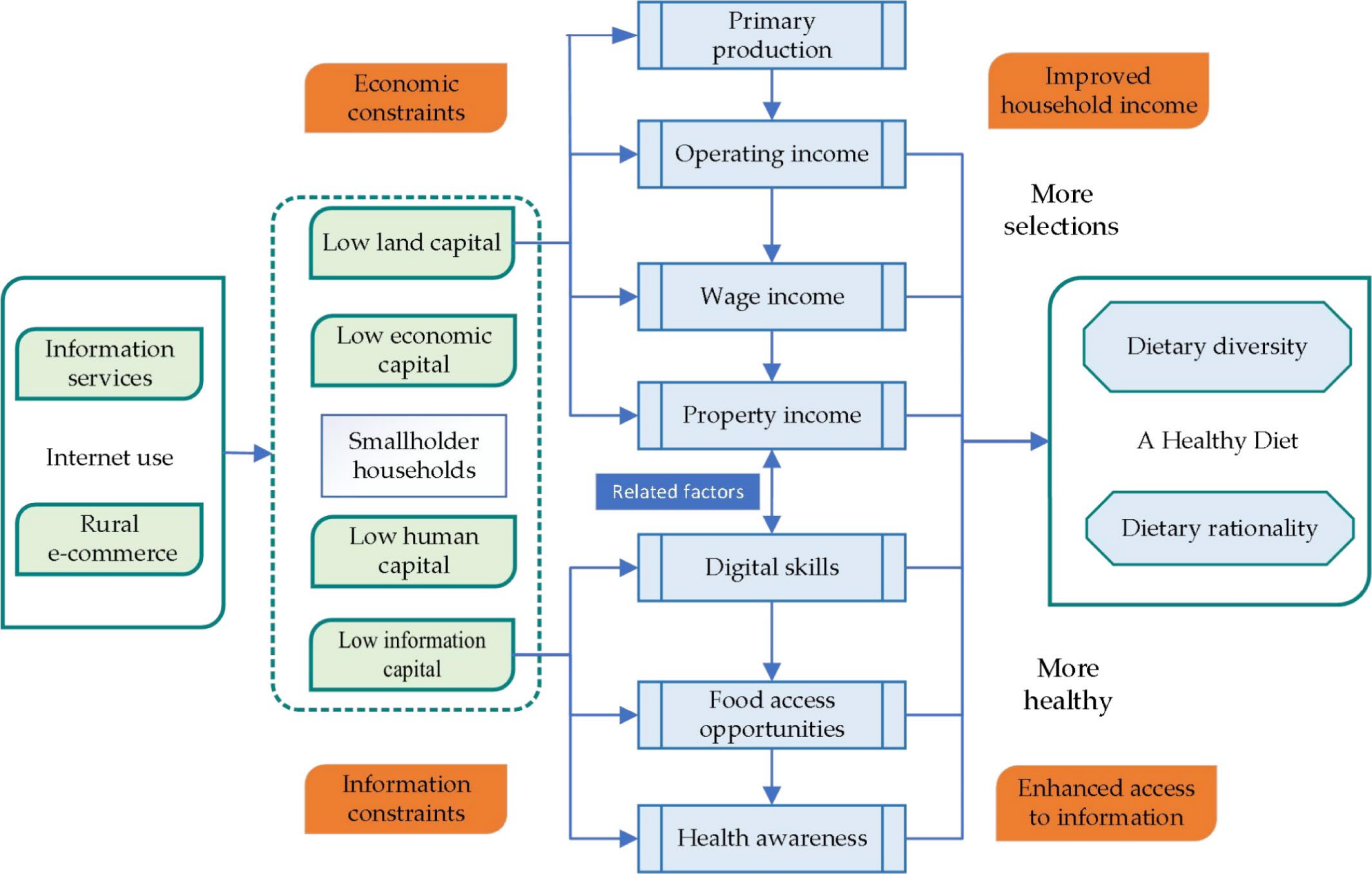
A theoretical model of the impact of Internet use on smallholder household diets.

Based on the above analysis, the theoretical analysis framework of this paper is shown in Figure 1.

## 3. Materials and methods

### 3.1. Data

The data in this article comes from the China Health and Nutrition Survey 2011 (CHNS).^1^ CHNS is an international collaborative cohort study conducted by the Population Center of the University of North Carolina at Chapel Hill and the Institute of Nutrition and Health (NINH) of the Chinese Center for Disease Control and Prevention. The database uses a multi-stage random sampling method to explore how China’s socioeconomic transition and family planning policies have affected the health and nutrition status of the population over the past 30 years, mainly in terms of continuous changes in disease patterns and health service status, and to provide a reference for policy adjustments in the new social era. As of now, CHNS is the only nationally representative database that can give precise details on Chinese citizens’ food consumption. Although the CHNS database has been updated to 2018, CHNS 2018 does not yet have the respondents’ comprehensive food consumption data (i.e., information on each food category). Therefore, only the CHNS data from 2011 may be used in this study and certain other recent investigations. In nine Chinese provinces (including Guangxi, Guizhou, Henan, Heilongjiang, Hubei, Hunan, Jiangsu, Liaoning, and Shandong), samples for CHNS were chosen using multi-stage random sampling. Our study could be well supported by the data in CHNS. China had a greater Internet penetration rate in 2011 than many other nations or regions had in 2019. As a result, despite the fact that our data is 10 years old, it still has a lot of research and policy significance for other developing economies. In order to obtain high-quality research data, the data were thoroughly screened. First, all urban household data were eliminated and only rural household registration data were retained, and only farming households with less than 3.33 hectares of arable land are retained. Second, the characteristic data of the household head corresponding to the “food procurement respondent” were matched and the individual data of non-household heads were eliminated. Third, the missing values, outliers, and other samples of important variables were deleted. Finally, a valid sample of 5,114 households was obtained.

### 3.2. Method

#### 3.2.1. Econometric model

There are two dependent variables in this paper: dietary diversity and dietary rationality. The dietary diversity is a continuous variable, so we constructed an OLS model to examine the effect of Internet use on dietary diversity. The OLS model is set up as in Formula (1):


(1) $$DDS_{i}=\alpha +\mathrm{\beta interne}t_{i}+\theta X_{i}+\varepsilon_{i}$$

Among them, $y_{i}$ is the dietary diversity score of farmers. ${\text{internet}}_{i}$ represents the Internet use of farm households, and $X_{i}$ indicates a series of control variables, mainly including family characteristics, village characteristics and individual characteristics.

The dietary rationality is a dummy variable, so we constructed a logistic regression model to examine the effect of Internet use on dietary rationality. The logistic model is set up as in Formula (2):


(2) $$\left\{ \begin{array}{c} \log\left(\frac{p}{1-p}\right)=\alpha +\mathrm{\beta interne}t_{i}+\theta X_{i}+\varepsilon_{i}\;\\ p=\mathrm{prob}\left(DRS_{i}=1\right) \end{array}\right.$$

Among them, $DRS_{i}$ is the dietary rationality status of farmers, $DRS_{i}$=1 represents households with a balanced dietary structure, and $DRS_{i}$=0 represents households with poor dietary structure.

#### 3.2.2. Propensity score matching (PSM)model

The propensity score matching method is a counterfactual inference method, the basic idea of which is to find a sample of controls similar to the treatment group to compare their effects, thus effectively solving the endogeneity problem arising from sample selection bias (73, 74). Because the dietary structure of farmers is a non-randomized experimental self-selection problem, it is highly susceptible to selective error, which can be effectively addressed by the PSM method. The specific steps are as follows:

Step 1. we used a logistic model to calculate the conditional probability of a household using the Internet, i.e., the propensity score.

Step 2. based on the propensity scores obtained through three methods: nearest neighbor matching, radius matching and kernel matching, we found a sample of farmers in the control group with propensity scores as similar as possible to those in the treatment group, in order to control and eliminate selectivity errors.

Step 3. PSM model requires that the variables used for matching meet the common support domain assumption and the balance test, and after the sample has been matched and the matching effect has been achieved, we calculate the average treatment effect (ATT). The ATT is calculated as shown below:

(3) $$PS_{i}=\mathrm{Pr}\left[D_{i}=1|X_{i}\right]=E\left[D_{i}=0|X_{i}\right]$$

(4) $$ATT=\frac{1}{N^{t}}\underset{i\in I^{t}\cap S}{\sum }\left\{Y_{i}-\underset{j\in I^{c}\cap S}{\sum }W_{ij}Y_{j}\right\}$$

Among them, $N^{t}$ is the number of samples, $I^{t}$ is the sample set of the disposal group (Using the Internet), $I^{c}$ is the sample set of the control group (Not using the Internet), $Y_{i}$ is the observed value of the sample of the disposal group, and $Y_{j}$ is the sample of the control group. The observations of j, S is the common support domain set, $W_{ij}$ is the matching weight, and ATT is the average disposition effect.

#### 3.2.3. Mechanism analysis model

In this section, we further analyze the pathways through which Internet use affect the dietary structure of farming households in terms of both raising income levels and enhancing access to information, to test the research hypotheses in the theoretical analysis section. Referring to the existing literature (86, 87), the mechanism model is set up as follows.


(5) $$\mathrm{ME}D_{i}=\alpha_{1}+\beta_{1}\mathrm{Skil}l_{i}+\theta_{1}X_{i}+\varepsilon_{i}$$

(6) $$Y_{i}=\alpha_{2}+\beta_{2}\mathrm{Skil}l_{i}+\theta_{2}X_{i}+\gamma MED_{i}+\varepsilon_{i}$$

Where $\mathrm{ME}D_{i}$ denotes mediating variables, including Includes effects of both raising household income (Income-sum) and enhancing access to information (Inf-enhance). Income-sum is measured by the logarithm of the total household income *per capita* of the farming household. Inf-enhance measures primarily whether farmers can access information through the Internet to meet their daily production and livelihood needs.

### 3.3. Variables

#### 3.3.1. Dependent variables

**Figure 2:**
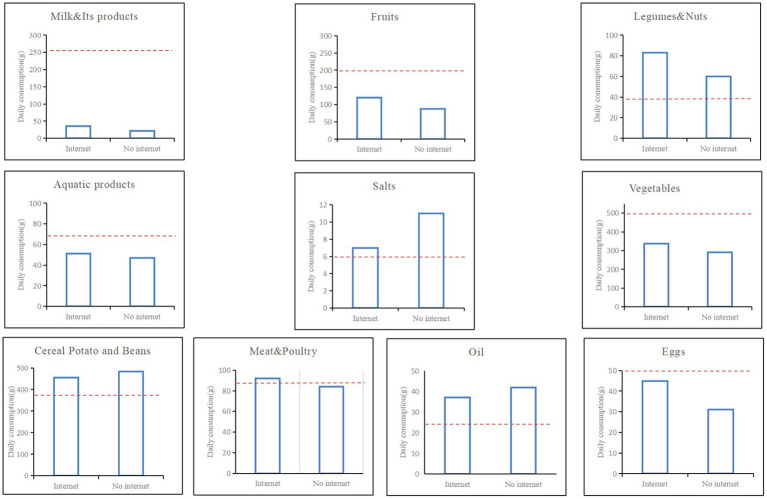
Average actual consumption of various food groups by smallholder households in China.

There are many indicators to measure the dietary quality, and this paper uses two of the most commonly used indicators with reference to the content of the CHNS survey, which are the dietary diversity score (DDS) and the dietary rationality status (DRS) respectively. DDS is the sum of all food varieties consumed by the farm household in the past week. A total of 10 food items were covered in the CHNS questionnaire, namely meat, fish and other aquatic products, fresh vegetables and fruits, dairy products, soya products, eggs, preserved foods, puffed/fried foods, and nuts, so DDS is a number ranging from 0 to 10. DRS is a dummy variable. We believe that it is difficult to scientifically measure dietary diversity and dietary balance with 1 day’s dietary data of farmers, and therefore, we choose 1 week’s dietary data as the dependent variable. If a family consumes both “fruits and vegetables” and “meat/eggs/fish” per week, DRS = 1; Otherwise, DRS = 0. In addition, the paper calculates the average values of various food items consumed by smallholder households, as shown in Figure 2 (The red line is the standard amount recommended by the 2016 Chinese Healthy Eating Guidelines).

It can be seen that in 2011, the average daily consumption of milk and its products, fruits, vegetables and eggs by smallholder households in rural areas of China did not meet the recommended standards of the Chinese dietary health guidelines, which is a preliminary indication that smallholder households generally have unhealthy diets. Compared to households without Internet, smallholder farmers with Internet have a clear advantage in consuming quality foods such as milk, vegetables, fruit and eggs, and tend to have lower oil and salt diets.

#### 3.3.2. Independent variables

The CHNS questionnaire asks each household member “do you/you use the internet.” Since this paper is looking at the Internet use of rural households, we set a dummy variable (Internet-skill) to measure whether the farming household has Internet use. If all members of the farm household do not use the Internet, Internet-skill = 0; Otherwise, Internet-skill = 1.

In order to examine the impact of different levels of Internet use, two additional indicators were added: the total number of household members using the Internet (Total-skill) and the total number participating in online shopping (Shopping-skill) (the relevant question in the CHNS questionnaire was “How much money did you/you spend on online shopping in the last 12 months,” households with values above zero are defined as participating in online shopping and those with values equal to zero are defined as not participating in online shopping). The marginal effect of Total-skill can be tested by the total number of Internet use in the household. In addition, the process of online shopping involves active search, real name authentication, and electronic payment, which requires a high level of user skills (75), and therefore Shopping-skill can be seen as a proxy variable for in-depth Internet use.

#### 3.3.3. Control variables

With reference to existing studies, this paper introduces three types of control variables, namely, variables on individual household head characteristics, variables on household characteristics, and variables on village characteristics (39, 76, 77). Household characteristics include gender, age, and education level; the number of household members and net household income *per capita*; village characteristics include village transportation conditions and economic status. The descriptive statistical characteristics of the specific variables are shown in Table 1.

**Table 1:** Definition and descriptive statistics of the variables involved.

Variables	Definition	Mean	SD
Dependent variables			
DDS	Dietary diversity score; Sum of household dietary items in the past week (0–10)	4.8566	1.9574
DRS	Dietary diversity status; 1 if the family diet in the past week included both meat, eggs, fish, vegetables and fruit, 0 otherwise	0.1257	0.3315
Independent variables			
Internet	1 if farming families use the Internet, 0 otherwise	0.1415	0.3486
Total	Total number of members using the Internet in the households (0–8)	0.7499	1.2306
Shopping	1 if farm households shopping online, 0 otherwise	0.1095	0.3123
Control variables			
Age	Age of the head of household (Years)	51.3744	13.205
Gender	Gender of head of household. Female = 1; male = 0	0.5586	0.4965
Education	Years of education of the head of household	5.8621	4.1454
Income	Logarithm of net household income *per capita*	8.6364	1.2595
Size	Family size, total number of members eating in the home (1–10)	3.3083	1.6904
Distance	Distance between the settlement and the county (km)	52.9143	40.824
Economic	Settled village economic status assessment	4.4053	1.3908
Inf-Enhance	How important do you think the Internet is as a channel for information (1–5)	1.5540	1.1509
IV	1 if family’s annual communication expenditure is greater than 1,000, 0 otherwise (RMB)	0.2849	0.4514
Observation	5,114		

## 4. Empirical results

### 4.1. Benchmark regression results

Table 2 reports the results of the benchmark regression of the impact of Internet use on the dietary structure of farm households. Columns (1) and (2) show the impact of farmers’ Internet use on dietary diversity and dietary rationality, respectively. The coefficients of the core explanatory variables are all significantly positive, indicating that Internet use can significantly improve dietary diversity and rationality among farm households, and thus improve dietary structure.

**Table 2:** Baseline regression results of the impact of Internet use on dietary diversity and dietary rationality of farm households.

variables	(1) DDS	(2) DRS	(3) DDS	(4) DRS	(5) DDS	(6) DRS
Internet	0. 5,391*** (0.1048)	0.7695*** (0.1394)				
Total			0.0652*** (0.0220)	0.1033*** (0.0366)		
Shopping					0.2812*** (0.0756)	0.4271*** (0.1155)
Age	0.0070*** (0.0023)	0.0051 (0.0042)	0. 0024 (0.0021)	−0.0037 (0.0038)	0.0023 (0.0021)	−0.0046 (0.0038)
Gender	0.0378 (0. 0546)	0. 1,171 (0.0979)	0. 0616 (0.0547)	0.1563 (0.0972)	0.0605 (0.0546)	0. 1,536 (0. 0972)
Income	0.1830*** (0. 0210)	0.1753*** (0.0413)	0. 1791*** (0.0212)	0.1701*** (0.0415)	0.1782*** (0.0211)	0. 1715*** (0. 0414)
Education	0.0602*** (0.0069)	0.1012*** (0. 0131)	0 0.0653*** (0.0068)	0.1110*** (0. 0129)	0 0.0640*** (0. 0068)	0 0.1106*** (0. 0129)
Size	0.0606*** (0. 0158)	−0.0023 (0.0283)	0.0452*** (0.0163)	−0.0274 (0.0293)	0.0510*** (0.0159)	−0.0203 (0.0287)
Economic	0.0038 (0.0199)	−0.0029 (0.0372)	0.0072 (0.0199)	0.0057 (0.0371)	0.0069 (0.0199)	0.0045 (0.0370)
Distance	−0.0031*** (0. 0007)	−0.0044*** (0.0013)	−0.0031*** (0.0007)	−0.0042*** (0.0013)	−0.0032*** (0.0007)	−0.0043*** (0.0013)
Province-FE	Yes	Yes	Yes	Yes	Yes	Yes
*N*	5,114	5,114	5,114	5,114	5,114	5,114
*R* ^2^	0.1580	0.1225	0.1537	0.1168	0.1546	0.1170

The standard error is shown in parentheses. *, ** and *** are statistically significant at 10, 5 and 1% levels, respectively. Province-FE represents provincial fixed effects. DDS refers to dietary diversity and DRS indicates dietary rationality.

Columns (3) and (4) report the marginal effects of dietary diversity and rationality per additional household member with Internet access, respectively; columns (5) and (6) report the effects of online shopping on dietary diversity and rationality for farm households, respectively. The results show that both the use of total Internet household members and online shopping can significantly increase the dietary diversity and dietary rationality of farming households, thus contributing to the upgrading of their dietary structure, which further validates the robustness of the findings.

The coefficients and signs of the control variables remain consistent with existing studies. The level of education of the household head and the net household income has a significant positive effect on the dietary structure of farm households. The distance from the settlement to the county town has a significant negative effect on the dietary structure of farm households.

### 4.2. Robustness tests

#### 4.2.1. Results of the IV regressions

The above baseline regressions may lead to endogeneity problems due to reverse causality, omission of variables, etc. and therefore require correction by the instrumental variable method. Specifically, the upgrading of farm households’ dietary structure may also affect their Internet use. In particular, farmers’ consumption of diversified food items such as meat, eggs, and milk has placed higher demands on freshness and quality and safety, which has led to the development of fresh food e-commerce and other industries, which not only requires farmers to acquire Internet use to track the execution of orders, but also requires farmers to further improve their skills to access information on the storage, preparation, and after-sale of highly nutritious food (78, 79). Farmers’ Internet use is not only an important guarantee for improving the quality of their meals, but also an effective way for them to access information efficient, so the higher the degree of a farmer’s dietary structure, the higher the degree of Internet use is required (80–82). Furthermore, although this paper controls for variables that may influence the upgrading of the dietary structure of farm households in the model setting, there may still be unobservable disturbances. Therefore, this paper attempts to mitigate the endogeneity problem by using an instrumental variable.

Referring the practice of He and Deng (83), the instrumental variable is selected as the 1-year communication expenditure of the farmer’s entire family. In contrast, communication costs belong to the category of consumption. From a broad perspective, the Internet also belongs to communication consumption, so the network usage habits in household communication expenditures will continue. Hence, endogenous variables in this study meet the relevant conditions. In theory, however, communication expenses will not directly affect farmers’ dietary structure.

The results of the IV regressions are reported in Table 3. The results show that the first stage *F*-values demonstrate that the instrumental variables selected for this paper are not weak instrumental variables. The results of the second-stage indicate that Internet use has a significant positive effect on dietary diversity and dietary rationality among farmers when endogeneity is addressed. Therefore, the conclusions of this paper are robust.

**Table 3:** Results of a 2-stage instrumental variable regression of the impact of Internet use on farm households’ diets.

	Internet-skill	DDS	Internet-skill	DRS
	First-stage	Second-stage	First-stage	Second-stage
Internet		0.4897*** (0. 1,081)		0.6203*** (0. 1,522)
IV	0.0773*** (0.0090)		0.0773*** (0.0090)	
Controls	Yes	Yes	Yes	Yes
Province FE	Yes	Yes	Yes	Yes
Wald F	72.76		54.71	
*N*	5,114	5,114	5,114	5,114

The standard error is shown in parentheses. *, ** and *** are statistically significant at 10, 5 and 1% levels, respectively. Province-FE represents provincial fixed effects. DDS refers to dietary diversity and DRS indicates dietary rationality.

#### 4.2.2. Results of the PSM model

Internet use is a self-selection process that may be influenced by their own endowments, and the structure of their diets may be influenced by these factors, thus leading to biased estimation results. The propensity score matching method can effectively solve the self-selection problem by matching farmers with and without Internet use, so that the two tend to be in a balanced and comparable state, and then comparing the dietary structure of farmers. In this paper, all control variables in the baseline model were selected as covariates. Given that different matching methods may lead to differences in model estimation results, five matching methods such as k-nearest neighbor matching were used in this paper for validation to ensure the reliability of the validation results, and the results are shown in Table 4. Where ATT represents the average processing effect for farm households with Internet use and ATE represents the average processing effect for all farm households. Average Treatment Effect (ATE) is a measure used to compare treatments or interventions in randomized trials of individuals, policy intervention assessments, and pharmaceutical trials. The average treatment effect measures the difference in the average outcome between individuals assigned to a treatment and control individuals. In randomized trials, the average treatment effect can be obtained by comparing the average outcome of the sample in treated and untreated individuals for estimation. For the dietary diversity of farmers, the coefficients of ATT are 0.1423 ~ 0.1733, and all coefficients are significantly positive, which indicates that farmers with Internet use increase the dietary variety by 14.23 to 17.33% compared to farmers without Internet use. For farmers’ dietary rationality, the coefficients of ATT are 0.0621 ~ 0.0639, and all coefficients are significantly positive, which indicates that farmers with Internet use increase the probability of having a balanced diet by 6.21 to 6.39% compared to farmers without Internet use. In addition, the coefficient on ATE was also significantly positive and similar to the corresponding ATT results. Thus, after accounting for self-selection, Internet use can still significantly contribute to dietary diversity and dietary rationality among farmers, and the estimates are highly consistent and robust.

**Table 4:** Results of a PSM robustness test of the impact of Internet use on farm households’ diets.

Matching method	Dietary diversity		Dietary rationality	
	ATT	ATE	ATT	ATE
Nearest neighbor matching	0.1668*** (0.0041)	0.1669*** (0.0040)	0.0639*** (0.0173)	0.0641*** (0.0180)
Radius matching	0.1423*** (0.0033)	0.1425*** (0.0030)	0.0633*** (0.0166)	0.0635*** (0.0167)
Kernel matching	0.1525*** (0.0043)	0.1526*** (0.0045)	0.0621*** (0.0156)	0.0623*** (0.0156)
Mahalas matching	0.1719*** (0.0045)	0.1720*** (0.0046)	0.0636*** (0.0141)	0.0637*** (0.0141)
Partial linear regression matching	0.1733*** (0.0051)	0.1736*** (0.0053)	0.0625*** (0.0136)	0.0627*** (0.0137)

The standard error is shown in parentheses. *, ** and *** are statistically significant at 10, 5 and 1% levels, respectively. ATT represents Average Treatment Effects on Treated. DDS refers to dietary diversity and DRS indicates dietary rationality.

In addition, using the nearest neighbor matching method, we further explore the impact of Internet use on the actual consumption of 10 food groups by smallholder households in China. The results are shown in Table 5. According to the findings, using the Internet increases smallholder families’ consumption of milk and its products by 2.9 grams, fruit consumption by 22.5 grams, egg consumption by 7.5 grams, and vegetable consumption by 27.1 grams. Internet use, on the other hand, reduced average daily use of oil and salt in rural smallholder families by 3.8 and 1.5 grams, respectively.

**Table 5:** Increased consumption of different categories of food by smallholder farmers with Internet.

ATT	(1) Milk and its products	(2) Fruits	(3) Legumes and nuts	(4) Meat and poultry	(5) Aquatic products
Internet	2.9217** (1.2601)	21.5299* (11.0927)	−9.7628 (8.1393)	7.1249 (7.0497)	0.5195 (0.4918)
ATT	(6) Cereal potato and beans	(7) Eggs	(8) Oil	(9) Vegetables	(10) Salts
Internet	−2.9217 (2.6019)	7.5299*** (2.0927)	−3.7628** (1.7393)	27.1249*** (7.0497)	−1.5195** (0.6918)

The standard error is shown in parentheses. *, ** and *** are statistically significant at 10, 5 and 1% levels, respectively. ATT represents Average Treatment Effects on Treated. DDS refers to dietary diversity and DRS indicates dietary rationality.

### 4.3. Results of the heterogeneity analysis

#### 4.3.1. Heterogeneity analysis based on educational level of household heads

China has a 9-year compulsory education system. Based on China’s school system, this paper classifies the years of education for heads of households into two categories, namely lower education group (0–9 years), and higher education group (more than 9 years). As shown in Table 6, Internet use can significantly improve dietary diversity and meal rationality among farmers with low levels of education; and have no impact on farmers with high levels of education.

**Table 6:** Results of heterogeneity analysis on the relationship between the Internet and the educational level of household heads.

Variables	DDS		DRS	
	Lower education (0–9 years)	Higher education (more than 9 years)	Lower education (0–9 years)	Higher education (more than 9 years)
Internet	0.5686*** (0.1049)	0.2069 (0.2469)	0.1013*** (0.0177)	0.0500 (0.0522)
Controls	Yes	Yes	Yes	Yes
Province FE	Yes	Yes	Yes	Yes
*N*	4,577	537	4,577	537
*R* ^2^	0.1514	0.1979	0.0784	0.2195

The standard error is shown in parentheses. *, ** and *** are statistically significant at 10, 5 and 1% levels, respectively. Province-FE represents provincial fixed effects. DDS refers to dietary diversity and DRS indicates dietary rationality.

The reason for this may be that the mastery of the Internet significantly enhances the learning and receptive abilities of farmers with low levels of education, which, in turn, contributes to the upgrading of their dietary structure, while farmers with higher levels of education have inherently stronger learning and information-seeking abilities, and the mastery of Internet use has relatively less impact on the upgrading of their dietary structure (84, 85).

#### 4.3.2. Heterogeneity analysis based on the age of household heads

Referring to the existing literature, this paper divides the sample of farm households into two groups: the youth group (household heads aged 18–60 years) and the elderly group (household heads aged 60 years and above), and removes the variable of household head’s age from the model. As shown in Table 7, Internet use have no significant impact on the upgrading of the diet of younger farmers (<60 years), but have a significant positive impact on the upgrading of the diet of older farmers (> = 60 years). This may be due to the influence of age. Older farmers pay more attention to a balanced and nutritious diet, and Internet use can provide farmers with access to food nutrition information and facilitate their increased intake of high-protein foods; young farmers, due to their relatively good health and not yet generally formed nutritional concepts, do not have a significant impact of Internet use on the upgrading of their dietary structure. This could mean that families with younger household head have already a good DDS and DRS and are more familiar with Internet so they have already adopted indications on a healthy diet.

**Table 7:** Results of heterogeneity analysis on the relationship between the Internet and the age of household heads.

Variables	DDS		DRS	
	Youth groups (18–59 years)	Elderly groups (more than 60 years)	Youth groups (18–59 years)	Elderly groups (more than 60 years)
Internet	0.6315 (0.5772)	0.5042*** (0.0891)	0. 0273 (0.0992)	0.1116*** (0. 0156)
Controls	Yes	Yes	Yes	Yes
Province FE	Yes	Yes	Yes	Yes
*N*	3,645	1,469	3,645	1,469
*R* ^2^	0.1495	0.1690	0.1219	0.1797

The standard error is shown in parentheses. *, ** and *** are statistically significant at 10, 5 and 1% levels, respectively. Province-FE represents provincial fixed effects. DDS refers to dietary diversity and DRS indicates dietary rationality.

#### 4.3.3. Heterogeneity analysis based on the household incomes *Per capita*

This paper classifies farm households into lower-income and higher-income groups based on their median *per capita* income levels, and removes the variable of *per capita* household income from the regression. As shown in Table 8, Internet use have a more significant pull on upgrading the dietary structure of higher-income farmers. High-income farmers have a significant financial advantage, and Internet use can provide farmers with more nutritional information, further increasing their consumption of foods such as meat, eggs and milk with higher nutritional value; while low-and middle-income farmers are constrained by their income levels, resulting in relatively little impact of Internet use on upgrading their dietary structure.

**Table 8:** Results of heterogeneity analysis on the relationship between the Internet and the incomes of farm households.

Variables	DDS		DRS	
	Lower-income	Higher-income	Lower-income	Higher-income
Internet	0.3500*** (0.0029)	0.6716*** (0.1345)	0.0905*** (0. 0205)	0.1116*** (0.00267)
Controls	Yes	Yes	Yes	Yes
Province FE	Yes	Yes	Yes	Yes
*N*	3,064	2,050	3,645	2,050
*R* ^2^	0.1101	0.1743	0.1510	0.1281

The standard error is shown in parentheses. *, ** and *** are statistically significant at 10, 5 and 1% levels, respectively. Province-FE represents provincial fixed effects. DDS refers to dietary diversity and DRS indicates dietary rationality.

### 4.4. Results of the mechanism analysis

As income growth is the basis for the upgrading of the dietary structure of farmers, this paper examines whether farmers’ Internet use contribute to the upgrading of their dietary structure by influencing their income from the perspective of income growth. Columns (1)–(3) in Table 9 reports the mediating effects of total farm household income on dietary structure. The results show that Internet use can significantly increase the income levels of farming households. Moreover, after controlling for total household income, Internet use can still significantly improve the dietary diversity and dietary rationality. Thus, Hypothesis 1 is verified.

**Table 9:** Results of mechanisms analysis of the impact of Internet use on smallholder diets.

Variables	(1) Income-sum	(2) DDS	(3) DRS	(4) Inf-enhance	(5) DDR	(6) DRS
Internet	0.2217*** (0.0600)	0.5299*** (0.0927)	0.7628*** (0.1393)	0.1249** (0.0497)	0.5195*** (0.0918)	0.7172*** (0.1402)
Income		0.1767 (0.1227)	0.1242*** (0.0382)			
Inf-enhance					0.2287*** (0.0261)	0.3522*** (0.0592)
Controls	Yes	Yes	Yes	Yes	Yes	Yes
Province FE	Yes	Yes	Yes	Yes	Yes	Yes
*N*	5,114	5,114	5,114	5,114	5,114	5,114
*R* ^2^	0.1090	0.1555	0.1580	0.1705	0.1653	0.1325

The standard error is shown in parentheses. *, ** and *** are statistically significant at 10, 5 and 1% levels, respectively. Province-FE represents provincial fixed effects. DDS refers to dietary diversity and DRS indicates dietary rationality.

Similarly, Internet use significantly enhance the accessibility of information to farmers, thus promoting dietary diversity and dietary rationality for farmers, as shown in column (4)–(6) of Table 8. Digital skills can expand farmers’ access to information on nutritional diets and health knowledge, enhance their ability to obtain information on rational diets, gradually form healthy dietary and nutritional concepts, and change their traditional eating habits of a single diet structure and excessive carbohydrate intake. With the enhancement of the ability to obtain nutrition and dietary information, the search cost for meat, eggs and milk will be greatly reduced, which will help farmers to increase their consumption of meat, eggs and milk, thus promoting the upgrading of their dietary structure. Thus, Hypothesis 2 is verified.

## 5. Discussion, conclusion, and implications

### 5.1. Discussions

In recent years, the incidence of chronic diseases such as cardiovascular and cerebrovascular diseases, diabetes and tumors has been on a rapid rise among farmers, which is closely related to the unreasonable dietary structure (88–90). Recently, the Chinese government is placing a high priority on nutritional health in rural areas (for example, in the “Report to the 20^th^ National Congress of the Communist Party of China,” one specifically mentioned in sections “to provide a higher level of food and nutritional needs in rural areas”). However, due to financial restrictions and a lack of nutritional health knowledge, many farmers have maintained traditional eating patterns (e.g., high-carb, high-salt, high-sugar diets and long-term consumption of food at night). Such a diet may have a significant influence on the welfare of rural people, resulting in increased sickness, increased medical burden, diminished human capital, and so on, endangering the sustainable growth and rehabilitation of rural communities (10, 11). In this context, this study found that the emergence of the Internet can improve dietary diversity and dietary rationality in rural areas, thereby upgrading the dietary structure, which provides a reference for the Chinese government and other developing countries to build new nutritional health promotion mechanisms.

The data from the current research encourages facilitating a relationship between the existing Internet technology and dietary health. The Internet changed the world and our lives. The “Metcalf rule” of the Internet has a more significant impact on developing countries. For instance, Hong et al. found that there is a significant positive association between Internet use and fast-food consumption among children in rural China (91). Similarly, Hassan et al. reported that nutrition knowledge from the Internet can significantly improve the dietary quality of Iranian youth (92). This study further verifies that there are income bracket differences in the impact of Internet use on dietary structure. The role of Internet use in high-income farm households in improving dietary structure is significantly greater than that in low-income farm households. Equally, in low-income farm households, dietary diversity and dietary rationality are lower than in high-income farm households, similar to the conclusions of Natalia et al. (93).

As income growth is the basis for farmers’ food consumption upgrading, this paper examines whether farmers’ Internet use contributes to their food consumption upgrading by affecting their income from the perspective of income growth. We found that Internet use has a positive association with the household income levels of smallholder farmers, thus contributing to the upgrading of their food consumption. The Internet use may expand the channels for smallholder farmers to increase their incomes, helping them to connect effectively with the larger market and to increase the sales price of their products while expanding the range of products sold, thus achieving higher levels of income. In addition, the use of the Internet offers smallholder farmers the possibility of obtaining more off-farm employment opportunities and optimizing asset allocation, contributing to higher-income levels and thus promoting increased consumption of meat, eggs and milk by smallholder farmers.

In addition, accessibility of information is not only relevant to smallholder farmers’ concerns about food nutrition, but also has an important impact on the formation of smallholder farmers’ perceptions of nutritional value. The results show that the use of the Internet has a significant positive impact on the accessibility of information to smallholder farmers, thus promoting their consumption of meat, eggs and milk. The Internet can expand the access to information on nutrition, diet and health for smallholder farmers, enhance their ability to obtain information on rational diets through Internet searches, and gradually form healthy dietary and nutritional concepts, thus changing their traditional diet of a single structure and excessive carbohydrate intake. With the enhancement of the ability to obtain nutrition and dietary information, the search cost for meat, eggs, and milk will be greatly reduced, which is conducive to the increase of meat, eggs, and milk consumption by farmers, thus promoting the upgrading of food consumption by farmers.

Finally, the findings of this study are somewhat different from those of existing studies. Ma et al. found that the Internet use significantly increased the consumption amounts of milk and its products, fruits, eggs, and vegetables (39). Lin et al. found that there is a strong correlation between problematic Internet use and poor dietary patterns of university students (94). However, there are less studies focusing on the impact of Internet use on the dietary quality in rural areas. This study found that Internet use can promote the upgrading of farmers’ diets by raising their income levels and enhancing access to information. Therefore, while promoting farmers’ income, the government should also promote health awareness. Only a “two-pronged approach” can achieve the desired outcome of a healthy diets.

Additionally, there are certain limitations in this study that need to be explored in future research. The connection between Internet use and healthy diets may be dynamic. Future research can create data panels that can be followed in real time to examine dynamic interrelationships. Furthermore, since the Chinese government introduced the “broadband China” policy in 2013, the rate of Internet penetration in China’s rural areas has progressively grown. Future research might look at whether the findings of this study apply to other developing countries. Particularly noteworthy is that this study does not allow for inferring a cause-effect relationship but an “association” between the variables. The heterogeneity and the mechanism analyses have evidenced the relationship exists, so the results can be used as a basis to carry out a further study to verify the cause-effect mechanism, administering questionnaires or interviews specifically designed.

### 5.2. Conclusion and implications

In the digital age, the impact of the Internet on the health and lifestyle changes of the population has become a topical issue (95–97). This study uses survey data of Chinese farmers in 9 provinces of mainland China to quantitatively study the impact of Internet use on dietary quality, which not only expands the research on the factors and mechanisms influencing dietary health, but also provides an important reference for a comprehensive review of the impact of the Internet on improving the welfare of rural households. The main conclusions are as follows:

(1)Consideration for sample selection bias correction, heterogeneity treatment, and endogeneity correction, this study found that the Internet use can significantly improve the dietary diversity and the dietary rationality of smallholder households, thus improving their dietary quality.

(2)This also discovered that Internet use boosted consumption of milk and its products (2.9 g), fruits (21.5 g), eggs (7.5 g), and vegetables (27.1 g), while lowering consumption of salts (1.5 g) and oil (3.8 g).

(3)Heterogeneity analysis found that the impact of Internet use on dietary quality is more obvious for low education level, elderly and higher-income farming households.

(4)Internet use can promote the dietary quality of smallholder households by raising their income levels and enhancing information access skills.

The validated conclusions outlined above can contribute and assist in emerging policy enlightenment. Firstly, given that Internet use can significantly enhance rural inhabitants’ dietary quality, the Chinese government should continue to invest in Internet infrastructure to promote Internet accessibility and information services, especially in rural areas. Secondly, considering that Internet use can be effective in increasing the household income of rural residents, relevant government departments should use the Internet platform to enhance the effectiveness of farmers’ digital technology use and digital resource utilization, continuously improve the level of farmers’ human capital, increase their non-farm employment capacity and asset allocation efficiency, and ensure that farmers steadily increase their income through multiple channels. Finally, information access has an important impact on healthy diets for farmers. Policymakers should actively use Internet platforms and digital tools to build a rational diet guidance mechanism, focusing on the precise dissemination of information on nutrition science and dietary concepts to rural residents to promote the development of healthy dietary concepts among farmers, thereby promoting the upgrading of dietary structures.

## Data availability statement

The original contributions presented in the study are included in the article/supplementary material, further inquiries can be directed to the corresponding author.

## Author contributions

MY: conceptualization, methodology, validation, resources, data curation, and supervision. MY and ZZ: software. ZW and MY: formal analysis and writing—original draft preparation. ZW: investigation. ZZ and MY: writing—review and editing. MY, ZW, and ZZ: visualization. All authors have read and agreed to the published version of the manuscript.

## Funding

This research was financially supported by the National Social Science Fund of China (Grant/Award Number: 17XTQ011); the MOE Project of Key Research Institute of Humanities and Social Sciences in Universities of China (Grant/Award Number: 16JJD790063); the National Social Science Foundation Youth Project of China (Grant/Award Number: 16JJD79006321CJY044) and Chongqing Postgraduate Research Innovation Project (Grant No. CYB22271).

## Conflict of interest

The authors declare that the research was conducted in the absence of any commercial or financial relationships that could be construed as a potential conflict of interest.

## Publisher’s note

All claims expressed in this article are solely those of the authors and do not necessarily represent those of their affiliated organizations, or those of the publisher, the editors and the reviewers. Any product that may be evaluated in this article, or claim that may be made by its manufacturer, is not guaranteed or endorsed by the publisher.

## Footnotes

^1^https://www.cpc.unc.edu/projects/china/data, accessed on 9 October 2022.
